# Brown Tumor as an Index Presentation of Severe Vitamin D Deficiency in a Teenage Girl

**DOI:** 10.7759/cureus.2722

**Published:** 2018-05-31

**Authors:** Alpesh Goyal, Hiya Boro, Rajesh Khadgawat

**Affiliations:** 1 Endocrinology and Metabolism, All India Institute of Medical Sciences, New Delhi, IND; 2 Endocrinology, All India Institute of Medical Sciences, New Delhi, IND

**Keywords:** brown tumor, hyperparathyroidism, vitamin d deficiency, pseudofracture, osteomalacia, nutritional osteomalacia

## Abstract

Brown tumor is a non-neoplastic fibro-cystic expansile bone lesion caused by parathyroid hormone excess. It has been commonly described in patients with primary hyperparathyroidism and secondary hyperparathyroidism due to chronic kidney disease. However, it is very rare to encounter a brown tumor in the setting of nutritional vitamin D deficiency. We describe the case of a 16-year-old girl who presented with brown tumor-like lytic lesion of femur caused by severe longstanding vitamin D deficiency. Treatment with elemental calcium and cholecalciferol resulted in correction of hyperparathyroid state, with the resultant disappearance of the bony lesion and remarkable symptomatic improvement. Unnecessary orthopaedic intervention may be avoided using a high index of suspicion and performing targeted investigations in such cases.

## Introduction

Brown tumor, also called as osteitis fibrosa cystica is an area of localized bone resorption due to parathyroid hormone excess. Histologically, the lesion resembles a giant cell tumor and diagnosis can be missed if blood levels of calcium and parathyroid hormone are not measured. Brown tumors are regarded as a characteristic bony manifestation of primary hyperparathyroidism, however, they have also been described in hyperparathyroidism secondary to chronic kidney disease. Despite the high prevalence of vitamin D deficiency (VDD), there are only a few cases describing brown tumor secondary to hyperparathyroidism complicating longstanding VDD [1-5].

## Case presentation

A 16-year-old girl presented to us with complaints of progressively increasing pain in left thigh for two years. At the time of presentation, the pain was moderately severe in intensity, requiring analgesics on a regular basis. It used to worsen on ambulation, resulting in limitation of her activities of daily living. There was no history of any local swelling or redness. She denied history of local trauma preceding the onset of pain. There was no history to suggest malabsorption or use of anticonvulsant drugs or indigenous medications. She was born out of a non-consanguineous marriage with normal birth and developmental history, and none of the family members had history of bone disease. Nutritional history was notable in the form of only occasional intake of milk and dairy products. The family used to live in an overcrowded basement of a two-storeyed building where exposure to sunlight was inadequate. Besides, patient preferred to remain indoors, moving out briefly only during early morning and evening hours in her premorbid state. For these complaints, she was evaluated in an outside hospital and diagnosed to have an aneurysmal bone cyst of the left femur. She was advised to undergo surgical intervention for the same; however, the anxious family brought her to our center for a second opinion.

Examination revealed a young, lean female with the height of 163 cm, a weight of 40 kg and BMI of 15 kg/m2. She had proximal myopathy involving bilateral lower limbs and walked with an antalgic gait with waddling towards the left side. There were no evident deformities involving the long bones or spine. Rest general and systemic examination were unremarkable.

Laboratory investigations of the patient have been summarised in Table 1.

**Table 1:** Biochemical results of the patient at presentation

Investigation	Units	Normal values	Result
Blood urea	mg/dl	20-40	34
Serum creatinine	mg/dl	0.5-1.4	0.4
Serum calcium (total)	mg/dl	8.5-10.4	7.9
Serum PO4	mg/dl	3.5-5.0	2.8
Serum ALP	IU	240-840	1324
Serum albumin	g/dl	>3.5	4.2
Serum 25 (OH)D3	ng/ml	12-100	<4
Serum intact PTH	pg/ml	15-65	840

**Figure 1:**
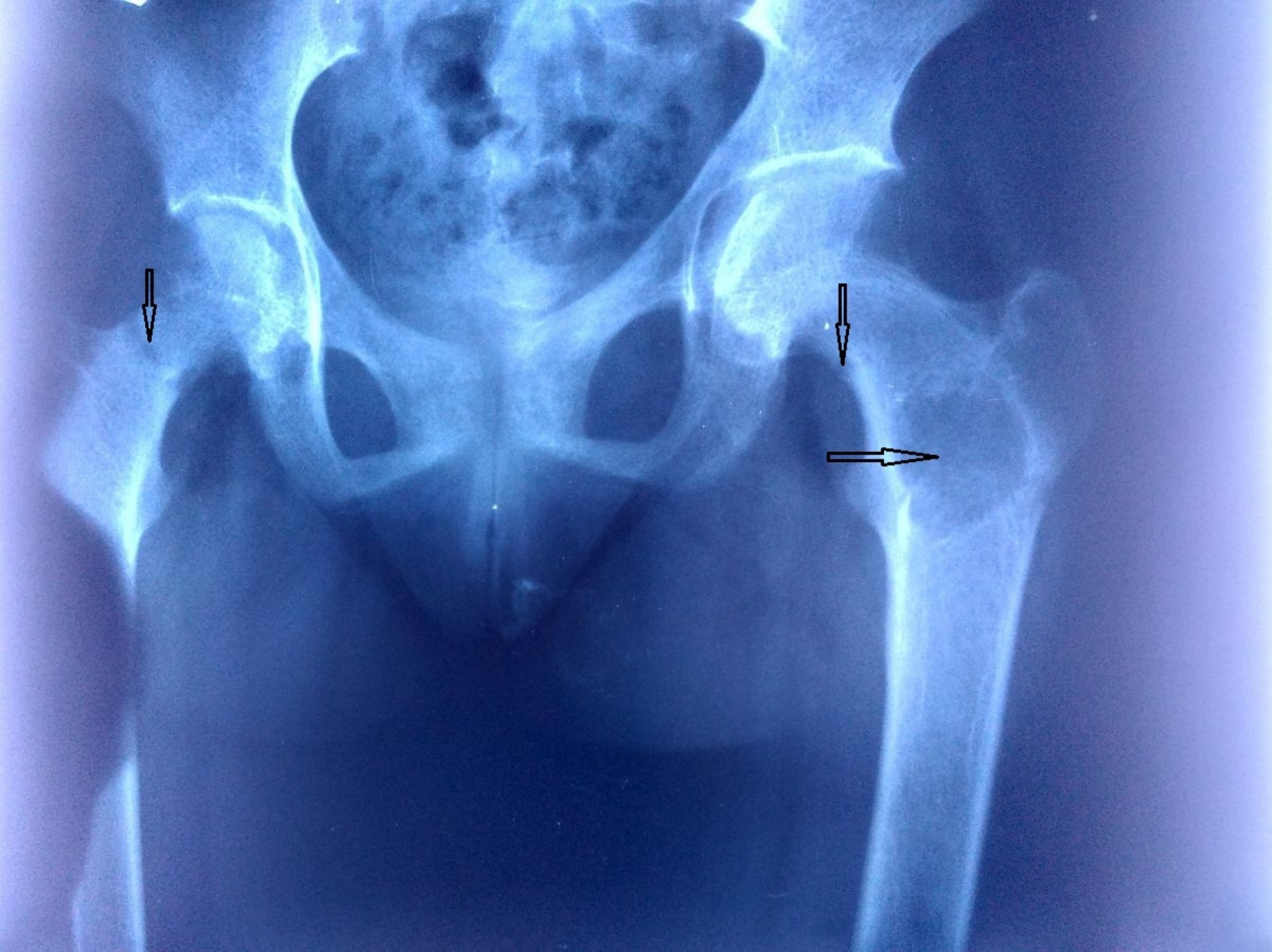
Pelvic radiograph at presentation An expansile lytic lesion involving left proximal femur (horizontal arrow) and pseudofractures involving bilateral femur neck (vertical arrows) can be seen on the pelvic radiograph. Also, note the widened joint space and blurrring of the symphysis pubis.

Total calcium was 7.9 mg/dl (normal 8.5-10.4), inorganic phosphorous 2.8 mg/dl (normal 2.5-4.5), alkaline phosphatase 1324 IU (normal 240-840), blood urea, creatinine, total protein, albumin, and liver function tests were normal. Tests for complete blood counts, fasting plasma glucose and urine routine examination were unremarkable. Serum intact parathyroid hormone was 840 pg/ml (normal 15-65), 25(OH)D3 <4 ng/ml (normal >20 ng/ml), total T4 8.2 ug/dl (normal 5.1-14.1 ), TSH 3.6 uU/ml (normal 0.27-4.2). X-ray pelvis revealed an expansile lytic lesion involving the trochanteric region of left femur, pseudofractures of bilateral femoral neck along with the widened joint space and irregular margins at the pubic symphysis (Figure 1).

Rest skeletal survey did not reveal any other lytic lesions or pseudofractures.

**Figure 2:**
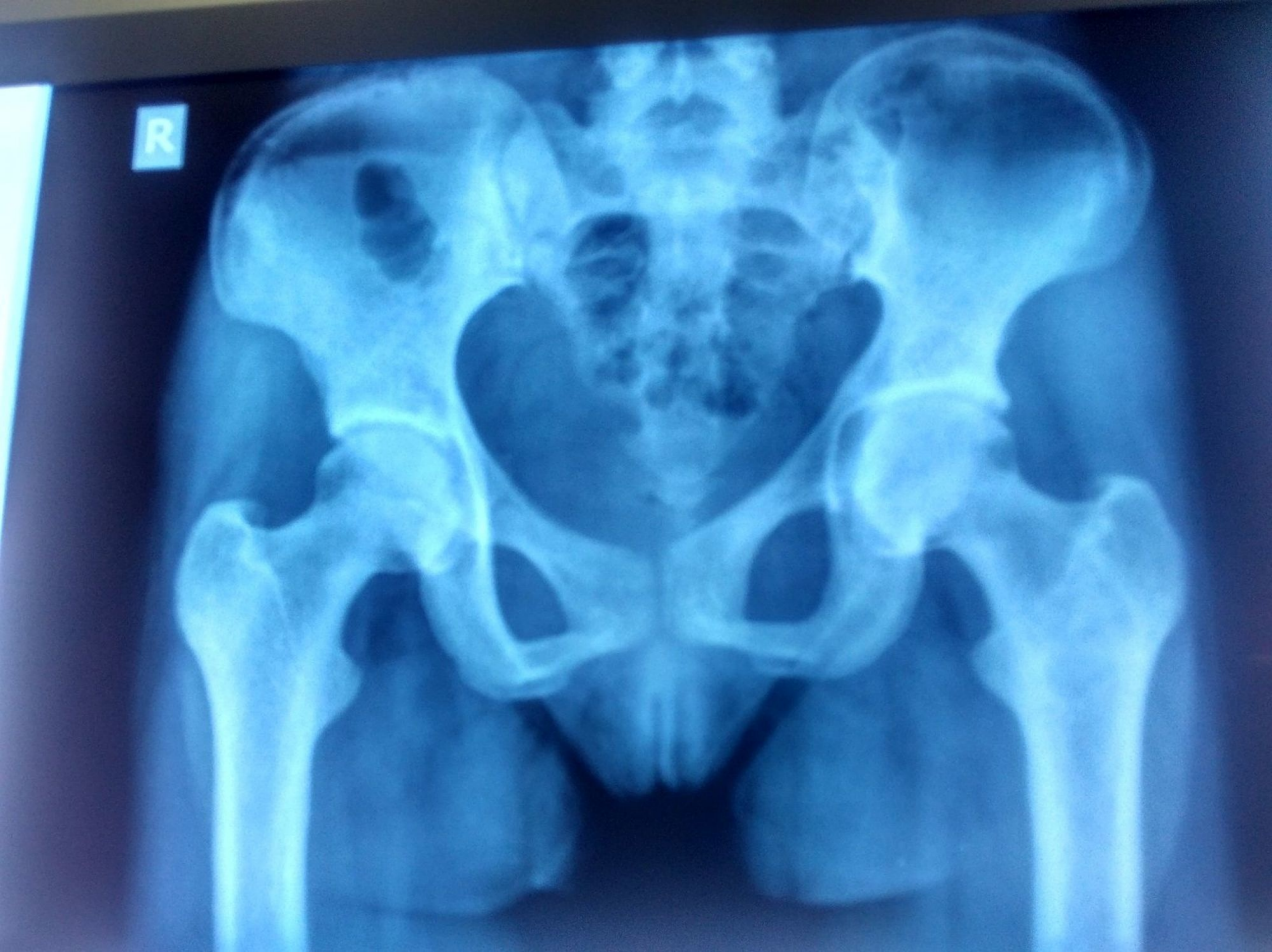
Pelvic radiograph 18 months after initial presentation Complete regression of the brown tumour and pseudofractures, with normalisation of the joint space and clearing of the pubic symphysis margins can be seen on the follow-up pelvic radiograph.

Diagnosis of nutritional osteomalacia with secondary hyperparathyroidism and an expansile lytic lesion of left femur, possibly brown tumor was entertained at this stage. She was started on one gram elemental calcium per day and vitamin D3 (cholecalciferol) 60,000 IU sachet once a week for eight weeks followed by once a month maintenance therapy. She was subsequently followed up at three monthly intervals. At follow-up visits, the patient reported significant improvement in pain and, by six months, she had returned to her premorbid functional sate. Biochemical parameters (Table 2) and radiology (Figure 2) also showed dramatic improvement with complete disappearance of the lytic lesion in association with correction of secondary hyperparathyroidism.

**Table 2:** Serial biochemical results of the patient

Investigation	Units	Normal values	Baseline	Follow up at 3 months	Follow up at 6 months	Follow up at 9 months	Follow up at 18 months
Blood urea	mg/dl	20-40	34	-	26	-	24
Serum creatinine	mg/dl	0.5-1.4	0.4	-	0.6	-	0.5
Serum calcium (total)	mg/dl	8.5-10.4	7.9	8.2	8.4	8.9	9.2
Serum PO4	mg/dl	3.5-5.0	2.8	3.4	4.0	4.3	4.4
Serum ALP	IU	240-840	1324	1009	567	345	183
Serum albumin	g/dl	>3.5	4.2	-	4.5	-	4.2
Serum 25 (OH)D3	ng/ml	12-100	<4	20	31	41	38
Serum intact PTH	pg/ml	15-65	840	184	174	183	41

Although tissue diagnosis was not available, the remarkable clinical and radiological improvement in association with correction of the hyperparathyroid state indicated that the lytic lesion was brown tumor related to parathormone excess.

## Discussion

Brown tumors are unifocal or multifocal bone lesions, characterized by increased osteoclast activity and fibroblast proliferation, encountered in patients with uncontrolled hyperparathyroidism. The term brown tumor is actually a misnomer, since these lesions are non-neoplastic. Histologically, the lesion is similar to a giant cell tumor, comprising of multinucleated giant cells in a background of spindle cell proliferation with large amounts of hemosiderin which imparts brown colour to the lesion. Although these lesions can occur at any skeletal site, the most common sites are the pelvis, ribs, long bone, clavicle, and mandible [6-7]. Radiological differentials include simple bone cyst, aneursymal bone cyst, giant cell tumor, fibrous dysplasia, osteomyelitis, and metastases [7]. Therefore, evaluation for serum calcium and intact parathormone should be performed in a patient presenting with expansile lytic lesions in these locations.

Brown tumors have been described at a frequency of 3%-4% in primary hyperparathyroidism and 1%-2% in secondary hyperparathyroidism in most series [6]. Brown tumors associated with hyperparathyroidism secondary to chronic kidney disease and nutritional VDD have been described in Table 3.

**Table 3:** Brown tumors associated with secondary hyperparathyroidism described in the literature

Study (Year)	Age (years), Sex	Etiology of Secondary Hyperparathyroidism	Location	Calcium (mg/dl)	PO4 (mg/dl)	ALP (IU)	iPTH (pg/ml)
1. Bereket et al. (2000)[1]	03, F	Nutritional VDD	Mandible	8.6	2.2	8986	844
2. Ahmed et al. (2006) [2]	17, F	Nutritional VDD	Pelvis, Rib	8.4	2.6	529	989
3. Bhadada et al. (2009) [3]	13, F	Nutritional VDD	Humerus	8.5	2.5	1263	92
4. Arunkumar et al. (2012) [4]	12, F	Nutritional VDD	Mandible	7.2	4.5	421	635
5.Yadav et al. (2014) [5]	12, M	Nutritional VDD	Mandible	7.5	2.8	13331	1459
6. Leal et al. (2006) [8]	31, F	CKD	Maxilla	8.4	7.1	1333	3086
7. Zwick et al. (2006) [9]	29, M	CKD	Frontal calvarium and orbital wall	9.6	7.3	-	450
8.Tarrass et al. (2008) [10]	18, M	CKD	Mandible	8.2	5.7	568	1335
9.Karabekmez et al. (2008) [11]	11, M	CKD	Maxilla & Mandible	8.4	9.7	1869	2528
10.Di Daniele et al. (2009) [12]	40, F	CKD	Maxilla	10.5	5.3	319	1700
11. Fatma et al. (2010) [13]	19, F	CKD	Mandible	9.1	6.2	2706	870
12. Fatma et al. (2010) [13]	37, F	CKD	Mandible	9.1	5.4	-	3687
13. Fatma et al. (2010) [13]	57, F	CKD	Maxilla	9.0	5.9	945	1500
14. Fatma et al. (2010) [13]	30, F	CKD	Mandible	8.5	7.1	2493	1115
15. Fatma et al. (2010) [13]	29, M	CKD	Maxilla	10.5	7.9	628	1450
16. Fatma et al. (2010) [13]	32, F	CKD	Maxilla	10.2	7.7	318	1142
17. Fatma et al. (2010) [13]	52, F	CKD	Maxilla	8.0	6.0	568	1700
18.Pinto et al. (2010)[14]	37, F	CKD	Maxilla and Mandible	-	-	1831	1927
19.Nabi et al. (2010) [15]	24, F	CKD	Maxilla and ipisilateral PNS	13.0	2.6	352	1591
20.Jakubowski et al. (2011) [16]	49, F	CKD	Mandible	-	-	-	-
21. Artul et al. (2013) [17]	46, F	CKD	Maxillary frontal bone	8.5	4.1	406	1282
22.Verma et al. (2014) [18]	31, F	CKD	Mandible	14.3	-	1963	234
23. Jafari-Pozve et al. (2014) [19]	29, M	CKD	Mandible, maxilla	8.7	6.3	2800	3552
24. Can et al. (2016) [20]	30, F	CKD	Mandible	9.1	4.2	1753	2930

In secondary hyperparathyroidism, the lesions have been described predominantly in the setting of end-stage renal disease with poorly controlled hyperparathyroid state [8-20]. Among these patients, craniofacial bones are the most common site affected. Management options in these patients include correction of underlying hyperparathyroid state using medications (like phosphate binders, active vitamin D analogues, calcium sensing receptor modulators), effective hemodialysis, renal transplantation or parathyroidectomy, in resistant cases. Despite nutritional VDD being highly prevalent in the general population, brown tumors due to resultant secondary hyperparathyroidism have been reported very rarely. There are only five such cases reported in the literature, predominantly in pediatric or adolescent age group. Bereket et al. [1] reported reversible brown tumor in mandible in a three-year-old girl in association with florid rickets secondary to nutritional VDD. Similarly, Ahmed et al. [2], Bhadada et al [3], Arunkumar et al [4] and Yadav et al [5] have described brown tumors involving craniofacial bones, humerus, pelvis and ribs in four separate reports in the setting of nutritional VDD. The cause for the rarity of brown tumors with VDD is not clear. A possible reason could be that treatment of VDD is fairly simple and patients are treated early and adequately, preventing a state of uncontrolled parathormone excess. On the other hand, poorly controlled hyperparathyroidism commonly occurs in the setting of end-stage renal disease despite best efforts. Clustering of VDD-related brown tumors in previously reported cases and our case in pediatric and adolescent age group could possibly be explained by the increased demands of calcium and vitamin D and hence susceptibility to VDD in this age group.

Due to the reversible nature, excision of brown tumor is not recommended unless the lesion causes compressive symptoms, significant cosmetic disfigurement or fails to regress on follow up. In our patient, the lesion healed completely with correction of secondary hyperparathyroidism using calcium and vitamin D supplementation. Healing of the lesion concurrent with improvement of metabolic parameters confirmed the diagnosis of brown tumor in this case. To the best of our knowledge, this is the first case reporting brown tumor-like lytic lesion in a long bone due to secondary hyperparathyroidism complicating longstanding VDD.

## Conclusions

We have described the case of a teenage girl, who presented with an expansile lytic lesion in the left femur, which was initially misdiagnosed as an aneurysmal bone cyst. Basic investigative work-up revealed nutritional VDD with secondary hyperparathyroidism, the treatment of which resulted in complete disappearance of the lesion. A basic metabolic work-up should be performed in all such cases and a therapeutic trial of calcium and vitamin D, followed by reassessment at 2-3 months may be done in indeterminate cases. Suspecting and investigating for secondary hyperparathyroidism is extremely important since appropriate treatment of the underlying metabolic state may prevent an unnecessary orthopedic intervention.

## References

[1] Bereket A, Casur Y, Firat P, Yordam N (2000). Brown tumor as a complication of secondary hyperparathyroidism in severe long-lasting vitamin D deficiency rickets. Eur J Ped.

[2] Ahmed M, Faraz HA, Almahfouz A (2006). A case of Vitamin D deficiency masquerading as occult malignancy. Ann Saudi Med.

[3] Bhadada SK, Padala RK, Bhansali A, Rao D, Mittal B (2009). Visual vignette. Endocr Pract.

[4] Arunkumar KV, Kumar S, Deepa D (2012). Brown tumor in mandible as a first sign of vitamin D deficiency: A rare case report and review. Indian J Endocr Metab.

[5] Yadav J, Madaan P, Jain V (2014). Brown tumor due to vitamin D deficiency in a child with cerebral palsy. Indian J Ped.

[6] Silverberg SJ, Bilezikian JP (2016). Primary hyperparathyroidism. Endocrinology: Adult and Pediatric [seventh edition].

[7] Olvi LG, Santini-Araujo E (2015). "Brown tumor: of hyperparathyroidism. Tumors and Tumor-Like Lesions of Bone.

[8] Leal CT, Lacativa PG, Gomes EM (2006). Surgical approach and clinical outcome of a deforming brown tumor at the maxilla in a patient with secondary hyperparathyroidism due to chronic renal failure. Arq Bras Endocrinol Metabol.

[9] Zwick OM, Vagefi MR, Cockerham KP, McDermott MW (2006). Brown tumor of secondary hyperparathyroidism involving the superior orbit and frontal calvarium. Ophthalmic Plast Reconstr Surg.

[10] Tarrass F, Benjelloun M, Bensaha T (2008). Severe jaw enlargement associated with uremic hyperparathyroidism. Hemodial Int.

[11] Karabekmez FE, Duymaz A, Keskin M, Tosun Z (2008). Huge deforming brown tumor of the maxilla and mandible in a patient with secondary hyperparathyroidism. J Plast Reconstr Aesthet Surg.

[12] Di Daniele N, Condò S, Ferrannini M (2009). Brown tumor in a patient with secondary hyperparathyroidism resistant to medical therapy: case report on successful treatment after subtotal parathyroidectomy. Int J Endocrinol.

[13] Fatma LB, Barbouch S, Fethi BH (2010). Brown tumors in patients with chronic renal failure and secondary hyperparathyroidism: report of 12 cases. Saudi J Kidney Dis Transpl.

[14] Pinto MC, Sass SM, Sampaio CP, Campos DS (2010). Brown tumor in a patient with hyperparathyroidism secondary to chronic renal failure. Braz J Otorhinolaryngol.

[15] Nabi Z, Algailani M, Abdelsalam M, Asaad L, Albaqumi M (2010). Regression of brown tumor of the maxilla in a patient with secondary hyperparathyroidism after a parathyroidectomy. Hemodial Int.

[16] Jakubowski JM, Velez I, McClure SA (2011). Brown tumor as a result of hyperparathyroidism in an end-stage renal disease patient. Case Rep Radiol.

[17] Artul S, Bowirrat A, Yassin M, Armaly Z (2013). Maxillary and frontal bone simultaneously involved in brown tumor due to secondary hyperparathroidism in a hemodialysis patient. Case Rep Oncol Med.

[18] Verma P, Verma KG, Verma D, Patwardhan N (2014). Craniofacial brown tumor as a result of secondary hyperparathyroidism in chronic renal disease patient: a rare entity. J Oral Maxillofac Pathol.

[19] Jafari-Pozve N, Ataie-Khorasgani M, Jafari-Pozve S, Ataie-Khorasgani M (2014). Maxillofacial brown tumors in secondary hyperparathyroidism: a case report and literature review. J Res Med Sci.

[20] Can O, Boynuegri B, Gokce AM (2018). Brown tumors: a case report and review of the literature. Case Rep Nephrol Dial.

